# Retraction Note: CircTLK1 promotes the proliferation and metastasis of renal cell carcinoma by sponging miR-136-5p

**DOI:** 10.1186/s12943-023-01764-4

**Published:** 2023-03-20

**Authors:** Jianfa Li, Chenchen Huang, Yifan Zou, Jing Ye, Jing Yu, Yaoting Gui

**Affiliations:** 1 Guangdong and Shenzhen Key Laboratory of Male Reproductive Medicine and Genetics, Institute of Urology, Peking University Shenzhen Hospital, Shenzhen-Peking University-the Hong Kong University of Science and Technology Medical Center, Shenzhen, 518000 China; 2 Anhui Medical University, Hefei, 230000 Anhui Province China; 3 Department of Urology, The Affliated Luohu Hospital of Shenzhen University, Shenzhen, 518000 China; 4 Department of Laboratory Medicine, Peking University Shenzhen Hospital, Shenzhen, 518000 China

**Retraction Note: Molecular Cancer 19, 103 (2020)**

**https://doi.org/10.1186/s12943-020-01225-2**

The Editor-in-Chief has retracted this article. Concerns were raised regarding a number of figures, specifically:

Figure 3c: the bottom left panel appears to partially overlap with the 769-P shNC panel of Figure 7h.

Figure 3d: the top left panel appears to partially overlap with the ACHN shrcireTLK1 panel in Figure 7j

Figure 7h: the ACHN shcireTLK1+CBx4 panel appears to partially overlap with the ACHN shNC panel in Figure 7j.

Additionally concerns were raised regarding the tumor sizes as displayed in Figure 8i. The Editor-in-Chief therefore no longer have confidence in the results and conclusions of this article.

Authors Jianfa Li, Chenchchen Huang, Jing Yu and Yaoting Gui disagree with this retraction. Authors Yifan Zou and Jing Ye have not responded to correspondence regarding this retraction.

